# *Lactobacillus delbrueckii *ssp. *bulgaricus *B-30892 can inhibit cytotoxic effects and adhesion of pathogenic *Clostridium difficile *to Caco-2 cells

**DOI:** 10.1186/1757-4749-1-8

**Published:** 2009-04-27

**Authors:** Pratik Banerjee, Glenn J Merkel, Arun K Bhunia

**Affiliations:** 1 Research and Development Division, LacPro Industries, LLC. 2020 E. Washington Boulevard, Suite 700, Fort Wayne, Indiana 46803, USA; 2 Department of Microbiology and Immunology, Indiana University School of Medicine, Fort Wayne, Indiana 46805, USA; 3 Molecular Food Microbiology Laboratory, Department of Food Science, Purdue University, West Lafayette, Indiana 47907, USA

## Abstract

**Background:**

Probiotic microorganisms are receiving increasing interest for use in the prevention, treatment, or dietary management of certain diseases, including antibiotic-associated diarrhea (AAD). *Clostridium difficile *is the most common cause of AAD and the resulting *C. difficile *– mediated infection (CDI), is potentially deadly. *C. difficile *associated diarrhea (CDAD) is manifested by severe inflammation and colitis, mostly due to the release of two exotoxins by *C. difficile *causing destruction of epithelial cells in the intestine. The aim of this study was to determine the effect of probiotic bacteria *Lactobacillus delbrueckii *ssp. *bulgaricus *B-30892 (LDB B-30892) on *C. difficile*-mediated cytotoxicity using Caco-2 cells as a model.

**Methods:**

Experiments were carried out to test if the cytotoxicity induced by *C. difficile-*conditioned-medium on Caco-2 cells can be altered by cell-free supernatant (CFS) from LDB B-30892 in different dilutions (1:2 to 1:2048). In a similar experimental setup, comparative evaluations of other probiotic strains were made by contrasting the results from these strains with the results from LDB B-30892, specifically the ability to affect *C. difficile *induced cytotoxicity on Caco-2 monolayers. Adhesion assays followed by quantitative analysis by Giemsa staining were conducted to test if the CFSs from LDB B-30892 and other probiotic test strains have the capability to alter the adhesion of *C. difficile *to the Caco-2 monolayer. Experiments were also performed to evaluate if LDB B-30892 or its released components have any bactericidal effect on *C. difficile*.

**Results and discussion:**

Co-culturing of LDB B-30892 with *C. difficile *inhibited the *C. difficile-*mediated cytotoxicity on Caco-2 cells. When CFS from LDB B-30892-*C. difficile *co-culture was administered (up to a dilution of 1:16) on Caco-2 monolayer, there were no signs of cytotoxicity. When CFS from separately grown LDB B-30892 was mixed with the cell-free toxin preparation (CFT) of separately cultured *C. difficile*, the LDB B-30892 CFS was inhibitory to *C. difficile *CFT-mediated cytotoxicity at a ratio of 1:8 (LDB B-30892 CFS:*C. difficile *CFT). We failed to find any similar inhibition of *C. difficile-*mediated cytotoxicity when other probiotic organisms were tested in parallel to LDB B-30892. Our data of cytotoxicity experiments suggest that LDB B-30892 releases one or more bioactive component(s) into the CFS, which neutralizes the cytotoxicity induced by *C. difficile*, probably by inactivating its toxin(s). Our data also indicate that CFS from LDB B-30892 reduced the adhesion of *C. difficile *by 81%, which is significantly (*P *<0.01) higher than all other probiotic organisms tested in this study.

**Conclusion:**

This study reveals the very first findings that *Lactobacillus delbrueckii *ssp. *bulgaricus *B-30892 (LDB B-30892) can eliminate *C. difficile*-mediated cytotoxicity, using Caco-2 cells as a model. The study also demonstrates that LDB B-30892 can reduce the colonization of *C. difficile *cells in colorectal cells. More study is warranted to elucidate the specific mechanism of action of such reduction of cytotoxicity and colonization.

## Background

Use of probiotic organisms to reduce and alleviate antibiotic-associated diarrhea (AAD) is receiving increasing interest in recent years [1-3]. *Clostridium difficile *is responsible for a potentially deadly bacterial infection and it is the most common etiologic agent of AAD or more precisely, a *C. difficile *associated diarrhea or colitis (CDAD or CDAC), resulting in severe diarrhea and inflammation [4,5]. The principal pathogenic determinants of *C. difficile *are two large exotoxins; A (TcdA) and B (TcdB), which are implicated for the clinical symptoms [6]. Traditionally, TcdA was considered to be the major component in eliciting the disease. More recently an equally important role of TcdB in pathogenesis has been demonstrated [6]. Cell death or cytotoxicity induced by Tcds is initiated by the glycosylation, followed by inactivation, of the small GTPases, Rho, Rac and Cdc-42 causing perturbations in the arrangement of actin cytoskeleton leading to cell death via apoptosis [7,8]. Along with Rho-GTPase dependent apoptosis event, Tcds are also reported to elicit apoptotic cell death via caspase-dependent pathways [7,9,10]. Disruption of the functionality of human and animal epithelial cell barriers by Tcds is believed to be caused by the above-mentioned mechanisms, where the toxins cause dysfunction of tight junctions in intestinal epithelia due to disaggregation of filamentous actin, resulting in cell detachment and rounding [8,11]. Inflammatory events in the intestine are also implicated as hallmarks of *C. difficile *infection (CDI) along with cytotoxicity, apoptotic and necrotic cell death [7]. Production of cytokines (interleukins 1–8, leukotrienes, histamine, etc.) and other inflammatory mediators from cells of the intestinal epithelial layer and lamina propria is triggered when TcdA or TcdB or both bind to receptors on intestinal epithelial cells [5,12-14]. The Tcds binding event results in the infiltration and activation of polymorphonuclear neutrophils (PMNs) [14,15]. Severe damage is done to villous enterocytes by PMN-derived inflammatory mediators, which act on these epithelial cells causing acute destruction and necrosis [14,15].

The incidence of CDI is increasing rapidly and is further complicated by the emergence of a more virulent, drug-resistant strain [4]. It is estimated that there are between 250,000 – 500,000 cases of CDI in the U.S. each year, resulting in longer hospital stays and an estimated $1.1 billion in additional costs [16,17]. Current treatment of CDAD, a regimen that has existed for the last 25 years, includes metronidazole or oral vancomycin along with discontinuation of the offending agent. Impaired colonization resistance frequently occurs following antibiotic therapy in hospitalized patients [18]. Emergence of new multi-drug resistant epidemic strains poses a great challenge to the effective treatment of CDI [19]. Along with conventional antibiotic therapy, administration of probiotic organisms to manage CDAD is drawing increasing attention [2]. Use of probiotic organisms such as *Saccharomyces boulardii *[20,21]; *Lactobacillus plantarum *299v [22,23]; *Lactobacillus rhamnosus *GG [24,25]; *Lactobacillus acidophilus *and *Bifidobacterium bifidum *[26] in conjunction with metronidazole or oral vancomycin therapy are reported in cases of CDAD [19-21]. Similarly, several reports of use of other probiotic organisms, individually and in combination with other strains, can be found in scientific reports in adult and infant AAD cases. These probiotic therapies include the use of *Lactobacillus rhamnosus *GG; *Bacillus clausii*; *Bifidobacterium longum*; *Clostridium butyricum *MIYAIRI; *Enterococcus faecium *SF68; *Lactobacillus acidophilus*; *L. acidophilus *and *L. bulgaricus *(Lactinex); *Lactobacillus acidophilus *and *Bifidobacterium longum*; *Lactobacillus acidophilus *and *Bifidobacterium lactis*; *Bifidobacterium lactis *and *Streptococcus thermophilus*; *Lactobacillus sporogenes *and fructo-oligosaccharide; *Lactobacillus acidophilus *and *Bifidobacterium infantis *[reviewed and analyzed in detail by McFarland, 2006 [27]]. The totality of evidence in these reports emphasizes the increased attention to probiotics and supports their inclusion as a choice of therapy, along with or in parallel with conventional antibiotic regimens, to prevent AAD or CDAD.

In the present study, we evaluated the efficacy of *Lactobacillus delbrueckii *ssp. *bulgaricus *B-30892, a probiotic bacteria found to be effective in inflammatory bowel disease as well as diarrhea, AAD, and CDAD [28,29], on *C. difficile-*mediated cytotoxicity on human enterocyte-like Caco-2 cell model.

## Methods

### Bacterial Strains and Culture Conditions

*Lactobacillus delbrueckii *ssp. *bulgaricus *B-30892 (LDB B-30892) was obtained from LacPro Industries, LLC, Fort Wayne, Indiana and was routinely grown anaerobically in Difco Lactobacilli MRS broth (Becton, Dickinson and Company [BD], Sparks, MD) or on MRS agar (BD) at 37°C. *Clostridium difficile *strain 9689 (CD-9689), a cytotoxin-producing strain, was purchased from the American Type Culture Collection (ATCC) and routinely grown anaerobically in Difco Reinforced Clostridial Medium (RCM; BD) or on RCM agar at 37°C. Six other commercially available probiotic and conventional lactobacilli strains; *L. delbrueckii *ssp. *bulgaricus *(LB-1 and LB-6), *L. acidophilus *(LB-2 and LB-3), and *L. casei *(LB-4 and LB-5 were isolated and biochemically characterized by API 50 CH system (bioMérieux, Hazelwood, MO) (Table 1). These lactobacillus cultures (LB-1 through LB-6, Table 1) were grown in MRS medium under the same conditions as LDB B-30892. In the present paper, *Lactobacillus delbrueckii *ssp. *bulgaricus *B-30892 is mentioned as LDB or LDB B-30892, *C. difficile *strain 9689 is mentioned as CD-9689 or CD and all other lactobacilli strains used were designated as LB or LB-1 through LB-6.

**Table 1:** List of different lactobacilli and *C. difficile *strains or isolates used

**Designation or Code**	**Strain**	**Source**
**LDB B-30892**	*L. delbrueckii *ssp. *bulgaricus *B-30892	LacPro
**LB-1**	*L. delbrueckii *ssp. *bulgaricus*	From commercial probiotic supplement
**LB-2**	*L. acidophilus*	From commercial probiotic supplement
**LB-3**	*L. acidophilus*	From Dr. A Bhunia, Purdue University
**LB-4**	*L. casei*	From commercial probiotic dairy drink
**LB-5**	*L. casei*	From Dr. A Bhunia, Purdue University
**LB-6**	*L. delbrueckii *ssp. *bulgaricus*	From commercial yogurt
**CD-9689**	*Clostridium difficile *strain 9689	ATCC

### Human Intestinal Cell Culture

The human intestinal epithelial cell line, Caco-2 (HTB-37), was purchased from the ATTC and cultured in Dulbecco's Modified Eagle medium (DMEM; Gibco, Invitrogen Corp.) with 20% fetal bovine serum (FBS). This cell line has been well established as a model to study the intestinal barrier function [30]. For the cytotoxicity assays, Caco-2 cells were grown to confluency in 96-well cell culture plates (Falcon, BD) at 37°C in 7% CO_2_.

### Cytotoxicity experiment

The effect of different lactobacilli on *C. difficile *induced cytotoxicity on a Caco-2 cell monolayer was tested using two groups of bacterial cell free supernatant (CFS) or bacterial conditioned medium (cell free) as described below:

#### CFS from co-culture

The bacterial strains were grown in 10 ml of the appropriate broth, anaerobically, at 37°C for 72 h. Twenty-four hours before the cytotoxicity experiment, one LDB B-30892 culture was centrifuged and the resulting pellet was resuspended into one *C. difficile *culture. Such lactobacilli cultures when added to *C. difficile *culture and grown together are designated as 'co-culture' in the present article. All bacterial cultures, controls and the mixed LDB B-30892/CD-9689 culture (LDB-CD co-culture), and mixture of other lactobacilli/*C. difficile *cultures (LB-1/CD-9689 through LB-6/CD-9689, Table 1, LB-CD co-culture) were then incubated anaerobically at 37°C for an additional 24 h. In some experiments, LDB B-30892 was heat-killed at 70°C for 45 min before centrifuging and resuspending in a CD-9689 culture. The CD-9689, LDB B-30892, and mixed lactobacilli/*C. difficile *cultures described above were centrifuged (10,000 *g*, 10 min, 5°C) and the supernatants collected and filter-sterilized (0.45 μm pore size). Thus several cell free supernatants were obtained from lactobacillus-clostridium co-cultures. The cytotoxicity of these filter-sterilized media, cell-free toxin preparation (CFT), and CFS containing lactobacilli 'factors' were tested on Caco-2 cultures as described below.

#### CFS mixture from individually grown cultures

In this case, we grew the lactobacilli (LDB and other LB-1 through LB-6) and *C. difficile *CD-9689 cultures individually under the conditions described above. All the different cultures (lactobacilli and *Clostridium*) were centrifuged (10,000 *g*, 10 min, 5°C) and the supernatants collected and filter-sterilized (0.45 μm pore size). Thus we obtained individual CFSs from all the eight bacterial cultures (Table 1). After this, CFS from LDB B-30892 (LDB-CFS) culture was added in different ratio to the crude toxin preparation from CD-9689 (CD-CFT) culture resulting in a cocktail of LDB-CFS + CD-CFT and tested for Caco-2 cytotoxicity. Similarly, the CFS obtained from six other commercially available probiotic and conventional lactobacilli strains, LB-1 through LB-6, designated as LB-CFS were mixed individually with CD-CFT and tested for cytotoxicity.

On the day of the cytotoxicity assay, the cell culture medium was removed from the Caco-2 monolayers and replaced with fresh cell culture media or various dilutions (1:2 through 1:2048 into DMEM, 20% FBS) of sterile RCM, sterile MRS, or the cell free supernatants (CFS or CFT) samples from various organisms (prepared as described above, either by co-culturing or obtained from separately grown cultures). The Caco-2 cultures were then incubated for 24 h. Cytotoxicity effects were assessed microscopically by examining the Caco-2 cell monolayers in multiwall plates with an inverted microscope as described below.

#### Photomicroscopic Analysis of Cytotoxic Effects

For photomicroscopy, similar cytotoxicity experiments as described above were conducted, except the Caco-2 cells were grown to confluency in Lab-Tek II Chamber Slide Systems (Nalge Nunc International Corp., Naperville, IL). Following treatment with toxin or antitoxin preparations for 24 h, the monolayers were washed once with PBS (20 mM, pH 7.2), fixed with 10% formalin in PBS for 10 min, the media chambers removed, and the slides were mounted with coverslips and Gel/Mount mounting medium (Biomeda Corp., Foster City, CA). Microscopic images were captured using a Leica DMRXA2 phase contrast microscope equipped with a Spot RTKE digital camera and software.

#### Live-Dead Analysis using Fluorescence Microscopy

We deduced the efficacy of LDB B-30892 in protecting Caco-2 cells from cytotoxic effect of CD-CFT, and compared that with other lactobacilli by a live-dead assay using two fluorescence dyes, propidium iodide (PI, red, dead cell indicator) and acridine orange (AO, green, live cell indicator), as described previously [31].

Briefly, a cell staining solution containing 20 μg/ml of AO and 100 μg/ml PI (Sigma) was prepared in sterile de-ionized water. The Caco-2 cells (exposed to toxins or anti-toxin preparations, as described in previous sections) were dislodged by trypsin treatment. Aliquots of 100 μl of cell suspension (1 – 2 × 10^6^/ml) were mixed with 100 μl of staining solution and analyzed immediately with a fluorescence microscope (Leica, model DMLB, Wetzlar, Germany, with SPOT software, version 4.6.4.2, Diagnostic Instruments, Sterling Heights, MI, USA), using green (for AO) and red filters (for PI). The detection of live (L), and dead (D) cells were done in the following manner, green (live), red (necrotic), both by visual scoring on a hemacytometer and by using image analysis software, SPOT, version 4.6.4.2 (image acquisition) and ImageJ v1.38 (NIH, USA) with "color counter" (v2001) and "color histogram" plug-ins (v2007) to analyze the images.

### Adhesion Assay

The goal of the adhesion assay was to investigate the ability of the cell free conditioned medium (CFSs) obtained from LDB B-30892 and other lactobacilli to modulate the adhesion of CD-9689 to the intestinal epithelial cell monolayer *in vitro*. The experimental set-up for this was similar to the detoxification experiment using Caco-2 cell as model. The adhesion assay was done as described previously [32-34] with some modification. In short, Caco-2 cells (1.1 × 10^4 ^cells/ml) at the post-confluence stage were seeded on Lab-Tek II Chamber Slide Systems (Nalge Nunc International, USA). After incubation for 14 days at 37°C in 7% CO_2_, the slides were washed with sterile PBS buffer and the test bacterial suspension of CD-9689 (with multiplicity of infection of 100 *C. difficile *cells to one Caco-2 cell; the *C. difficile *suspension medium in this case was a 1:1 volume/volume RCM-lactobacilli CFS) was added to each chamber with a Caco-2 monolayer. A 1:1 volume/volume RCM-MRS (without any lactobacillus conditioning) served as the control. The chambered slides were incubated at 37°C in 7% CO_2 _for 2 h. After incubation, Caco-2 cells were washed four times with PBS and then immersed in PBS for 30 s. Cells were immersed in 1.0 ml of 100% ethanol for 5 min, air dried, and immersed in 1.0 ml of Giemsa staining solution (2.5 ml of KaryoMAX Giemsa staining solution [Invitrogen Corp., Carlsbad, Calif.] and 48.5 ml of 10 mM potassium phosphate buffer [20 mM KH_2_PO_4_, 20 mM K_2_HPO_4_; pH 6.8]), washed twice with distilled water, air dried again, and examined under a Leica DAS Mikroskop at a magnification of ×1,000. Counts of bacterial adhesion were taken at four to five random locations for a total of at least 150 Caco-2 cells, averaged, and statistically analyzed by the Duncan test by using SAS software (SAS Institute, Cary, N.C.). The inhibition of adhesion was calculated by taking the adhesion of CD-9689 without any treatment as 100 percent by the formula described.

### Growth inhibition assay

Co-cultures of *C. difficile *(CD-9689) and *Lactobacillus *(LDB B-30892) on solid medium as well as in broth were done. In one such study, CD-9689 was streaked on RCM agar and cross-streaked with LDB B-30892. In some other experiments, CD-9689 and LDB B-30892 were co-cultured in RCM broth followed by selective plating on RCM and MRS agar plates, respectively. To study if the LDB-CFS caused any growth inhibition on CD-9689 cells, different ratios of LDB-CFS were added to RCM containing CD-9689 cells. All plates and tubes were incubated anaerobically at 37°C.

### Statistical Analysis

Data are expressed as mean ± Standard Error of Mean (SEM). Statistical analyses of data were performed using GraphPad Prism (version 3.02, GraphPad Software, San Diego, CA). Comparisons of cytotoxicity values between single control and CFS or CFT exposed sample means were made using two-tailed Student's *t *test or Tukey's pairwise comparison test. The limit for statistical significance was set at *P *< 0.05. Results of inhibition of adhesion assay were statistically analyzed by the Duncan test by using SAS software (SAS Institute, Cary, N.C.).

## Results and discussion

### Diminished cytotoxic effect of *Clostridium difficile *(CD-9689) in presence of *Lactobacillus delbruckeii *ssp. *bulgaricus *B-30892 on Caco-2 cells

**Figure 1:**
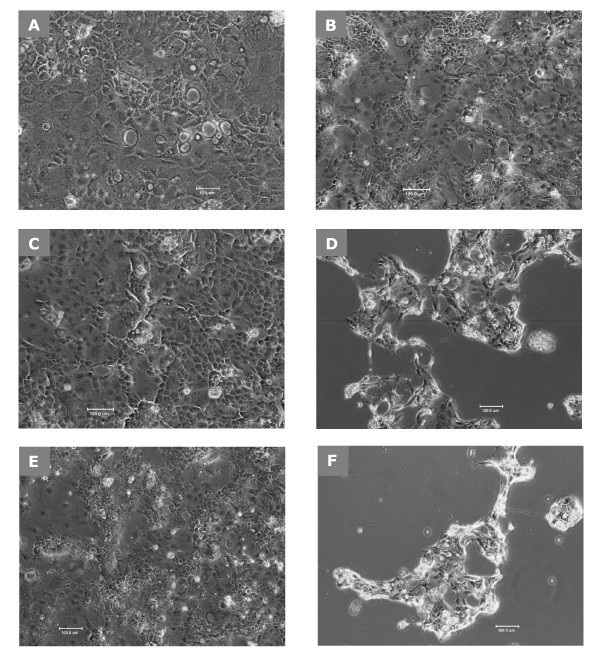
**Role of LDB B-30892 on CD-9689 mediatedcytotoxicity**. Confluent Caco-2 cultures were incubated 24 h in DMEM containing 20% FBS. Cell free supernatants (CFS or CFT) from different treatments were added to the cell monolayer. (A) Caco-2 control cells (200× original magnification); (B) Normal-appearance after 24 h of incubation with fresh RCM diluted 1:4 into DMEM containing 20% FBS; (C) Appearance of Caco-2 in LDB B-30892 CFS, diluted 1:4 into DMEM, 20% FBS. (D) Caco-2 cultures incubated 24 h in cell-free RCM after growing CD-9689 24 h in it. Note the cytopathic effect of the CD-9689 cytotoxin. (E) Caco-2 cultures incubated 24 h in cell-free RCM after growing CD-9689 and LDB B-30892 24 hours together in it. (F) Caco-2 cultures incubated 24 h in cell-free RCM after growing CD-9689 for 24 h.

*C. difficile*-CFT causes a significant cytopathic effect on Caco-2 cells, presumably because of the production of extracellular cytotoxin(s) by this strain. Figure 1A shows the normal appearance of Caco-2 cells (untreated control). Caco-2 cells remain healthy following treatment with RCM (Figure 1B) and anti-toxin preparation from LDB B-30892 (LDB-CFS) (Figure 1C). While *C. difficile *cytotoxin after incubation with CFT from CD-9689-conditioned RCM for 24 h caused cytopathic effect (rounding and cell detachment) on Caco-2 cells (Figure 1D). The lack of a cytopathic effect of CFT from CD-9689-conditioned RCM after co-culturing CD-9689 and LDB B-30892 together for 24 h on Caco-2 cells was observed (Figure 1E). In contrast, Figure 1F shows the cytopathic effect when no LDB B-30892 were co-cultured with CD-9689 (same as depicted in Figure 1D). We observed the similar cytotopathic effects (as in Figures 1D and 1F) when heat killed LDB B- 30892 were incubated with CD-9689 (data not shown).

The lack of a cytotoxic effect shown in Figure 1E provided clues that CFT from CD-9689-conditioned RCM was no longer cytotoxic after co-culturing with LDB B-30892, possibly due to detoxification of the *C. difficile *cytotoxin(s) by LDB B-30892 or factor(s) produced by this organism. To verify this effect, we preformed a semi-quantitative assessment of *C. difficile *cytotoxin activity and its detoxification by LDB B-30892.

Table 2 shows representative results of the semi-quantitative assessment of *C. difficile *cytotoxin activity and its detoxification by LDB B-30892. The *C. difficile*-conditioned medium was cytotoxic up to a dilution of 1:8, while the highest concentrations of RCM, MRS, or LDB B-30892-conditioned medium (1:2) was non-cytotoxic and maintained normal Caco-2 cellular morphologies. Importantly, the LDB B-30892/*C. difficile *co-culture supernatant was also non-cytotoxic at its highest concentration (1:2). This detoxification by LDB B-30892 required living cells (which are metabolically active during the co-culturing with CD-9689 for 24 h) since adding heat-killed LDB B-30892 cells with *C. difficile *resulted in cytotoxicity equivalent to that of the *C. difficile*-conditioned medium alone.

**Table 2:** Effect of sterile media and CFS/CFT from CD-9689 and LDB B-30892 co-culture on Caco-2 cells.

**Media only or CFS**	**Appearance of Caco-2 cells**			
	
Dilution	**1:2**	**1:4**	**1:8**	**1:16**
RCM	N^1^	N	N	N
CD-9689 CFS (CFT)	C^2^	C	C	N
MRS	N	N	N	N
LDB B-30892 CFS (LDB-CFS)	N	N	N	N
LDB B-30892 – CD-9689 co-culture CFS	N	N	N	N
Heat-killed LDB B-30892 – CD-9689 co-culture CFS	C	C	C	N

^1 ^N, normal cellular morphology

^2 ^C, signs of cytotoxicity (e.g. cell rounding and detachment from the substrate)

Based on these initial *in-vitro *observations, we further investigated the following two issues: (i) Does LDB B-30892 release a bioactive component in the CFS, which results in the detoxification of *C. difficile-*mediated cytotoxicity; and (ii) Is the detoxification of *C. difficile-*mediated cytotoxicity unique to LDB B-30892?

To test the first question, LDB B-30892 and CD-9689 were grown separately in MRS and RCM broth, respectively, under the conditions mentioned earlier. CFS from the LDB B-30892 culture was added in different ratios to CFT from the CD-9689 culture (in a similar manner as in the semi-quantitative assay described above). We found that CFS from LDB B-30892 culture inhibited the cytotoxic effects of CFT from CD-9689 culture when added at a ratio of 1:1 through 1:8, and this inhibitory effect diminished in ratios lower than 1:16. These results indicate that LDB B-30892-conditioned CFS contains a bioactive component(s) or in other words, LDB B-30892 releases one or more extracellular components in the growth medium, which were responsible for inhibiting or deactivating the exotoxins released by *C. difficile *(in growth medium), thus protecting Caco-2 cells from *C. difficile-*mediated cytotoxicity.

**Figure 2:**
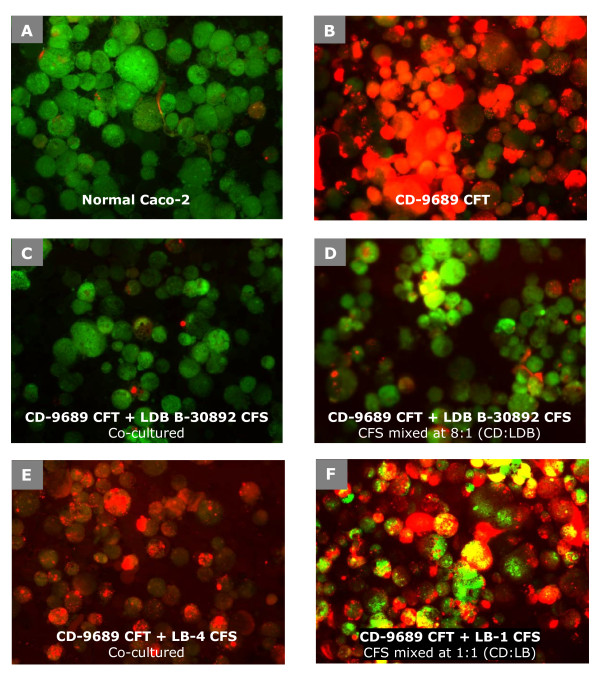
**Live and dead status of Caco-2 cells after exposures to CFT from CD-9689 with or without treatments with different lactobacillus CFS**. After the treatments with CFS or CFTs, Caco-2 cells were trypsinized and then florescence dye, PI and AO were added. A green fluorescence (AO) indicates live cell, while a red fluorescence (PI) indicates dead cell.

**Figure 3:**
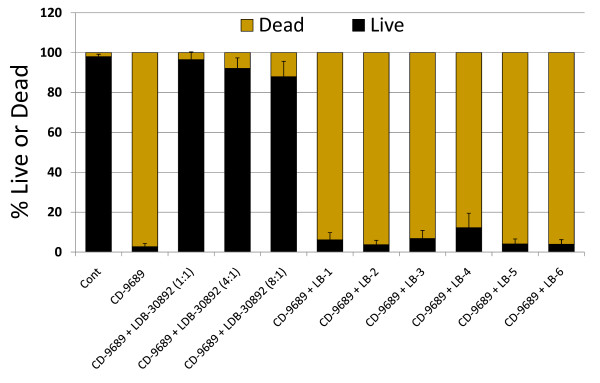
**Quantitative assessment of live and dead status of Caco-2 cells after exposures to CFT from CD-9689 with or without treatments with different lactobacilli CFS**. Cont, control without any treatment, CFS from LDB B-30892 was added to CD-9689 CFT so that the resulting ratio of CFT from CD and CFS from LDB are 1:1, 4:1 and 8:1. The ratio of CFS from other lactobacilli (LB-1 through LB-6) to CFT from CD-9689 were 1:1. Values are presented as Mean ± Standard error of mean (SEM) of three experiments done in duplicate.

So far, we observed that cell-free supernatant from co-cultured LDB/CD lacks cytotoxic property, also, when supernatants from individually grown culture of LDB B-30892 and CD-9689 were mixed together, the CFS and CFT mixture showed no cytotoxic effect. This observation indicates that CFS from LDB B-30892 resulted in the inhibition of cytotoxicity of CFT from CD-9689. To test whether the inhibitory property of the above-mentioned cytotoxicity of *C. difficile *is unique to LDB B-30892 or if it is typical to any lactobacillus, we tested six other commercially available probiotic or conventional lactobacillus strains (LB-1, LB-2, LB-3, LB-4, LB-5, LB-6; Table 1). CFS samples from the lactobacilli cultures (LB-1 through LB-6), either co-cultured with CD-9689 (LB/CD co-culture supernatant) or separately grown and then mixed together (LB/CD mixed CFS/CFT) were tested for cytotoxicity and compared with the results from LDB B-30892. We found no other lactobacillus tested was able to inhibit the cytopathic effect of CD-9689-conditioned CFT, in either co-culture or in CFS/CFT mixtures of LB/CD. We deduced the efficacy of LDB B-30892 to protect Caco-2 cells from the cytotoxic effect of CD-9689-conditioned CFT and compared that with other lactobacilli by a live-dead assay using fluorescence dyes propidium iodide (PI, red, dead cell indicator) and acridine orange (AO, green, live cell indicator) [31] (Figure 2 and Figure 3).

Figure 2A depicts control Caco-2 cells without any treatment; most cells are viable as they incorporated AO but not PI. Caco-2 cells treated with CFT from CD-9689 are shown in Figure 2B, where most of the cells were dead as indicated by the incorporation of PI by the vast majority of the cells. Figures 2C and 2D represent the protective effect of LDB B-30892-conditioned CFS on Caco-2 cells when co-cultured (Fig 2C) with CD-9689 or when separately cultured LDB-CFS was added to CFT from CD-9689 (Fig 2D). In both Figures 2C and 2D, the vast majority of the cells excluded PI (red fluorescence) indicating a higher live cell population. However, no other lactobacilli tested in the present study were able to detoxify CD-9689-conditioned CFT as similarly demonstrated by LDB B-30892 CFS. Figures 2E and 2F show the inability of LB-4, a *L. casei *isolated from a commercial probiotic yogurt and LB-1, a *L. delbrueckii *ssp. *bulgaricus *isolated from a probiotic supplement, to inhibit the cytotoxicity induced by CFT from CD-9689, as depicted by higher PI counts indicating that the majority of cells are dead.

Figure 3 shows the counts of live and dead Caco-2 cells after exposure to CFT from CD-9689, with or without treatments with different lactobacilli CFS. A mixture of cell-free extracts from CD-9689/LDB B-30892 at a ratio of 1:1 resulted in less than 4% cell death, while the mixture at a ratio of 4:1 caused only 12% cell death. No other probiotic organisms tested could result in such protection of Caco-2 cell death when the probiotic CFS aliquots were mixed to CD-9689-CFT.

The lack of a cytotoxic effect of CD-9689 in the presence of LDB B-30892-conditioned CFS on Caco-2 cells is thought to be due to a possible proteolytic activity of the antitoxic component released by LDB B-30892. Indeed, a similar detoxification of the *C. difficile *toxin A and B by a protease produced by *Saccharomyces boulardii *has been reported [35,36].

### *Lactobacillus delbruckeii *ssp. *bulgaricus *B-30892 can inhibit the adhesion of *Clostridium difficile *to human colonic cells

**Figure 4:**
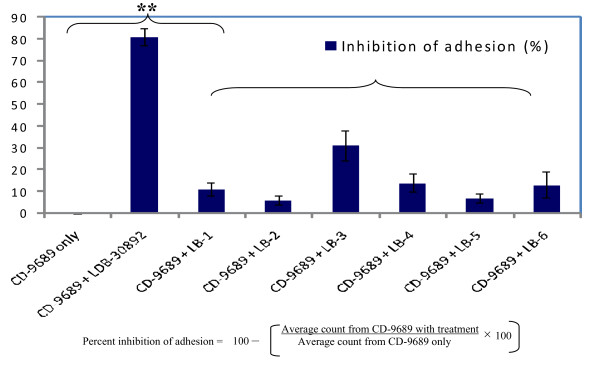
**Inhibition of CD-9689 to Caco-2 monolayer by cell free supernatants from different lactobacilli**. CD-9689 cells were suspended in 1:1 volume/volume RCM- lactobacillus conditioning MRS at a MOI of 100: 1 and incubated at 37°C in 7% CO_2 _for 2 h. After incubation, Caco-2 cells were washed, stained with Giemsa and counted. The above formula was used to calculate the percent inhibition of adhesion CD-9689 to Caco-2 monolayer. Values are presented as Mean ± Standard error of mean (SEM) of three experiments done in duplicate. ** *P *< 0.01.

The ability of different lactobacilli strains to inhibit the adhesion of enteropathogens varies significantly. For example, Ingrassia et al. [37] reported the ability of *L. casei *DN-114 001 to inhibit the adhesion of adherent-invasive *Escherichia coli *isolated from Crohn's disease patients. On the contrary, Gueimonde *et al*. (2006) reported that *L. casei *TMC 0409 actually increased the adhesion of *C. diffcile *ATCC 9689 (CD-9689) in a Caco-2 model. These apparent contradictory results can be found regularly in probiotic or gut microbiology literature. It is widely accepted that commensal or probiotic organisms prevent colonization of enteropathogens on the gut epithelial surface by competitive exclusion. Sharing of common carbohydrate-binding sites in probiotic organisms allows the blocking of adhesin receptors, thus promoting the inhibition of pathogen adhesion by steric hindrance [32,38]. Apart from whole cell bindings, another prominent mechanism which is believed to play a crucial role are the soluble factors (such as loosely adhered surface proteins of certain lactobacilli) released in the gut lumen which may cause the inhibition of adhesion or colonization of enteropathogens [38,39]. We were interested to investigate whether CFSs from different lactobacilli including LDB B-30892 contain such soluble factor(s) that may reduce the adhesion of CD-9689. It is evident from Figure 4, that culturing *C. difficile *(CD-9689) with CFS from LacPro's probiotic *L. delbrueckii *spp.*bulgaricus *(LDB B-30892) significantly reduced the adhesion of this pathogen to the Caco-2 cell monolayer. No other lactobacilli CFS tested showed an inhibition of adhesion of CD-9689 as efficient as LDB B-30892.

### *Lactobacillus delbruckeii *ssp. *bulgaricus *B-30892 did not inhibit the growth of *Clostridium difficile*

**Figure 5:**
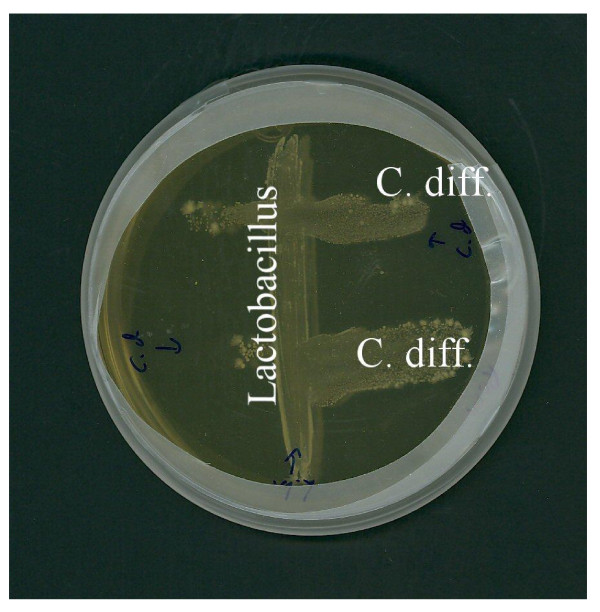
**Failure of LDB B-30892 (Lactobacillus) to inhibit the growth of CD-9689 (*C. diff*)**. LDB B-30892 and CD-9689 were co-cultured on RCM agar anaerobically at 37°C for 48 h. LDB B-30892 and CD-9689 were co-cultured on TSA anaerobically at 37°C for 48 h.

To investigate if the whole cell or CFS from LacPro's probiotic *L. bulgaricus *(LDB B-30892) were able to inhibit the growth of pathogenic *C. difficile *(CD-9689), we did a co-culture experiment on solid medium (trypticase soy agar, TSA) as well as in broth (RCM). Our result of growth inhibition study reveals that neither LDB B-30892 nor CFS from LDB B-30892 inhibited the growth or viability of CD-9689 (Figure 5). Since the CD-9689 tested grew well in presence of LDB B-30892, it appeared that no soluble and diffusible growth-inhibiting substances were released from this bacterium, as has been reported for another probiotic *Lactobacillus *[40]. Although, recent reports have shown that *L. casei *inhibits the infection of Caco-2 cells by *S*. Typhimurium [41] and *L. rhamnosus *blocks epithelial barrier disruption by *E. coli *O157:H7 [42] without producing any growth-inhibiting substances.

## Conclusion

In the present study, we have gathered initial evidences which indicated that cell-free supernatant (alternatively, the conditioned medium) from probiotic *L. bulgaricus *LDB B-30892 reduced the cytotoxicity produced by pathogenic *C. difficile *ATCC 9689. At the same time, the adhesion of *C. difficile *was reduced significantly (*P *< 0.01) by LDB B-30892. We hypothesize that one or more bioactive component(s) is released by LDB B-30892 in its growth medium (CFS), which is (or are) the probable causative agent(s) of inhibition of cytotoxins, i.e., detoxification and inhibition of adhesion which may occur via several possible mechanisms, such as, proteolytic cleavage of toxin or toxin receptors, blockage of toxin receptors or *C. difficile *adhesion molecules on host cells by competitive binding by the bioactive agent(s). However, the specific mechanism of the detoxification and diminished adhesion of *C. difficile *in presence of CFS from LDB B-30892 remains to be elucidated. In future, we plan to purify the bioactive component(s) from LDB B-30892-CFS, further characterize and investigate the underlying mechanism of reduced cytotoxicity and adherence of *C. difficile *on Caco-2 cells.

## Competing interests

PB works as a paid employee for LacPro Industries. Experimental work conducted for this study at the laboratories of GJM and AKB was funded by LacPro Industries.

## Authors' contributions

PB, GJM and AKB designed the study. PB and GJM carried out the experiments. PB coordinated the study and wrote the manuscript. All authors read and approved the final manuscript.
